# Supplementary material for: Cell type-specific gene regulatory network inference from single cell transcriptomics with ctOTVelo
Source: bioRxiv. 2026 Mar 14:2026.03.11.711174. Preprint. [Version 1] doi: 10.64898/2026.03.11.711174 (PMC13060787; doi:10.64898/2026.03.11.711174)
Supplement: Supplement 1 [file NIHPP2026.03.11.711174v1-supplement-1.pdf]

bioRxiv preprint doi: https://doi.org/10.64898/2026.03.11.711174; this version posted March 14, 2026. The copyright holder for this preprint (which was not certified by peer review) is the author/funder, who has granted bioRxiv a license to display the preprint in perpetuity. It is made available under aCC-BY 4.0 International license.

A Notation

We utilize notation consistent with OTVelo:

Table S1: Table of Notations

Notation	Description
$g \in \{1, .., m\}$	Index for genes
$t \in \{t_1, ..., t_N\}$	Index for time points
$n(t) \in \mathbb{R}$	Number of cells at time t
$c_t = \{1, 2, ..., n(t)\}$	Index for cell
$\mathbf{X} = (\mathbf{X}^{t_1}, \mathbf{X}^{t_2}, ..., \mathbf{X}^{t_N})$	Time-stamped gene expression
$\mathbf{X}^t \in \mathbb{R}^{c_t \times g}$	Gene expression at time t
$x_g^{c,t} \in \mathbb{R}$	gene expression of gene g for cell c at time t
$D^{t,\tilde{t}} \in \mathbb{R}^{n(t) \times n(t)}$	Difference between cells at time t and $\tilde{t}$
$D_{c,\tilde{c}}^{t,\tilde{t}} \in \mathbb{R}^{n(t) \times n(t)}$	Entry of $D^{t,\tilde{t}}$ representing Euclidean distance between cells c and $\tilde{c}$
$S^t \in \mathbb{R}^{n(t) \times n(t)}$	Distances between cells at time t of a k-nearest neighbors graph, where $k = \min\{50, 0.2n(t), 0.2n(\tilde{t})\}$
$\ell_{GW} : \mathbb{R}^2 \to \mathbb{R}^+$	Function that accounts for misfit pairwise distances
$\alpha \in \mathbb{R}$	Hyperparameter for weighting the Wasserstein and Gromov-Wassserstein terms
$T \in \mathbb{R}^{n(t) \times n(t)}$	Coupling matrix learned from entropic fused Gromov-Wasserstein optimal transport
$\prod(p, q) = \{T : T\mathbf{1} = p, T^{\mathrm{T}}\mathbf{1} = q, T \geq 0\}$	Family of coupling matrices given source marginal density p and target marginal density q
$v_g(x^{t,c}, t) \in \mathbb{R}$	Velocity of gene g for cell c at time t

B Full quantitative metrics for human hematopoiesis

Table S2: Mean Top k edges F1 score (± SD) for global GRNs against UniBind

Top K edges	ctOTVelo	OTVelo	SINCERITIES	GRNBoost2	scMTNI
100	**5.12e-04 ± 0.00e+00**	3.56e-04 ± 0.00e+00	<u>3.70e-04 ± 1.70e-04</u>	3.14e-04 ± 4.19e-05	3.38e-04 ± 4.62e-05
200	**1.04e-03 ± 0.00e+00**	<u>8.41e-04 ± 0.00e+00</u>	<u>7.15e-04 ± 3.26e-04</u>	6.54e-04 ± 7.83e-05	6.89e-04 ± 1.06e-04
300	**1.60e-03 ± 0.00e+00**	<u>1.29e-03 ± 0.00e+00</u>	1.06e-03 ± 4.63e-04	9.85e-04 ± 1.07e-04	1.03e-03 ± 1.28e-04
400	**2.11e-03 ± 0.00e+00**	<u>1.79e-03 ± 0.00e+00</u>	1.44e-03 ± 6.20e-04	1.32e-03 ± 8.44e-05	1.43e-03 ± 1.95e-04
500	**2.58e-03 ± 0.00e+00**	<u>2.19e-03 ± 0.00e+00</u>	1.79e-03 ± 7.54e-04	1.72e-03 ± 7.50e-05	1.80e-03 ± 2.70e-04
1000	**4.89e-03 ± 0.00e+00**	<u>4.60e-03 ± 0.00e+00</u>	3.50e-03 ± 1.54e-03	3.51e-03 ± 1.41e-04	3.55e-03 ± 4.71e-04
2000	**9.43e-03 ± 0.00e+00**	<u>9.28e-03 ± 0.00e+00</u>	7.02e-03 ± 1.48e-03	6.92e-03 ± 1.91e-04	6.64e-03 ± 7.90e-04

Table S3: Mean Top k edges F1 score (± SD) for global GRNs against Cus_ChIP_2

Top K edges	ctOTVelo	OTVelo	SINCERITIES	GRNBoost2	scMTNI
100	**3.46e-04 ± 0.00e+00**	1.48e-04 ± 0.00e+00	<u>2.92e-04 ± 4.48e-04</u>	1.53e-04 ± 2.88e-05	2.63e-04 ± 1.19e-04
200	**7.65e-04 ± 0.00e+00**	2.72e-04 ± 0.00e+00	<u>5.68e-04 ± 9.27e-04</u>	3.50e-04 ± 8.17e-05	5.54e-04 ± 1.31e-04
300	**1.28e-03 ± 0.00e+00**	4.44e-04 ± 5.42e-20	8.04e-04 ± 1.31e-03	5.13e-04 ± 7.86e-05	<u>8.35e-04 ± 2.27e-04</u>
400	**1.67e-03 ± 2.17e-19**	6.16e-04 ± 0.00e+00	1.06e-03 ± 1.73e-03	6.60e-04 ± 8.15e-05	<u>1.13e-03 ± 2.87e-04</u>
500	**2.09e-03 ± 0.00e+00**	8.61e-04 ± 0.00e+00	1.31e-03 ± 2.10e-03	8.16e-04 ± 1.13e-04	<u>1.43e-03 ± 3.00e-04</u>
1000	**4.13e-03 ± 0.00e+00**	1.98e-03 ± 0.00e+00	2.02e-03 ± 2.99e-03	1.94e-03 ± 1.45e-04	<u>3.02e-03 ± 4.35e-04</u>
2000	**7.99e-03 ± 0.00e+00**	4.30e-03 ± 0.00e+00	3.42e-03 ± 4.09e-03	4.10e-03 ± 1.62e-04	<u>5.63e-03 ± 6.00e-04</u>

bioRxiv preprint doi: https://doi.org/10.64898/2026.03.11.711174; this version posted March 14, 2026. The copyright holder for this preprint (which was not certified by peer review) is the author/funder, who has granted bioRxiv a license to display the preprint in perpetuity. It is made available under aCC-BY 4.0 International license.

Table S4: Mean Top k edges F1 score (± SD) for global GRNs against Cus_KO_0.01

Top K edges	ctOTVelo	OTVelo	SINCERITIES	GRNBoost2	scMTNI
100	**8.53e-04 ± 0.00e+00**	7.31e-04 ± 0.00e+00	6.34e-04 ± 2.02e-04	6.82e-04 ± 9.75e-05	7.33e-04 ± 1.77e-04
200	**1.46e-03 ± 0.00e+00**	**1.46e-03 ± 0.00e+00**	1.13e-03 ± 3.30e-04	1.35e-03 ± 1.86e-04	1.30e-03 ± 2.63e-04
300	2.12e-03 ± 0.00e+00	**2.48e-03 ± 0.00e+00**	1.70e-03 ± 4.13e-04	1.95e-03 ± 1.40e-04	2.04e-03 ± 3.45e-04
400	2.84e-03 ± 0.00e+00	**3.62e-03 ± 0.00e+00**	2.43e-03 ± 6.96e-04	2.69e-03 ± 2.49e-04	2.70e-03 ± 4.06e-04
500	3.61e-03 ± 0.00e+00	**4.46e-03 ± 0.00e+00**	3.01e-03 ± 8.86e-04	3.42e-03 ± 2.03e-04	3.37e-03 ± 4.20e-04
1000	7.41e-03 ± 0.00e+00	**8.24e-03 ± 0.00e+00**	6.22e-03 ± 2.11e-03	6.55e-03 ± 4.10e-04	6.72e-03 ± 7.06e-04
2000	**1.49e-02 ± 1.73e-18**	1.49e-02 ± 0.00e+00	1.17e-02 ± 2.62e-03	1.25e-02 ± 3.47e-04	1.30e-02 ± 9.45e-04

Table S5: Mean Top k edges F1 score (± SD) for global GRNs against Cus_KO_0.01_Cus_ChIP_2_intersect

Top K edges	ctOTVelo	OTVelo	SINCERITIES	GRNBoost2	scMTNI
100	**5.38e-03 ± 0.00e+00**	2.99e-03 ± 0.00e+00	2.87e-03 ± 2.66e-03	3.59e-03 ± 6.55e-04	5.27e-03 ± 1.28e-03
200	8.71e-03 ± 0.00e+00	6.97e-03 ± 0.00e+00	5.34e-03 ± 5.39e-03	6.62e-03 ± 2.53e-03	**1.01e-02 ± 3.34e-03**
300	1.18e-02 ± 0.00e+00	1.02e-02 ± 0.00e+00	9.03e-03 ± 9.33e-03	9.37e-03 ± 3.36e-03	**1.28e-02 ± 3.29e-03**
400	1.48e-02 ± 0.00e+00	1.15e-02 ± 0.00e+00	1.21e-02 ± 1.20e-02	1.22e-02 ± 2.58e-03	**1.64e-02 ± 4.43e-03**
500	1.76e-02 ± 0.00e+00	1.23e-02 ± 0.00e+00	1.51e-02 ± 1.32e-02	1.34e-02 ± 2.25e-03	**1.96e-02 ± 3.57e-03**
1000	2.59e-02 ± 3.47e-18	1.65e-02 ± 0.00e+00	**3.45e-02 ± 1.70e-02**	1.34e-02 ± 2.25e-03	3.18e-02 ± 4.54e-03
2000	3.55e-02 ± 0.00e+00	2.55e-02 ± 0.00e+00	**5.53e-02 ± 1.83e-02**	1.34e-02 ± 2.25e-03	5.08e-02 ± 3.03e-03

Table S6: Mean Top k edges F1 score (± SD) for global GRNs against Cus_KO_0.01_union_intersect

Top K edges	ctOTVelo	OTVelo	SINCERITIES	GRNBoost2	scMTNI
100	**1.21e-02 ± 0.00e+00**	**1.21e-02 ± 0.00e+00**	9.21e-03 ± 5.54e-03	1.03e-02 ± 1.63e-03	6.06e-03 ± 9.58e-04
200	2.26e-02 ± 0.00e+00	**2.40e-02 ± 0.00e+00**	1.67e-02 ± 1.11e-02	1.95e-02 ± 2.66e-03	1.27e-02 ± 2.79e-03
300	**2.95e-02 ± 3.47e-18**	2.67e-02 ± 3.47e-18	2.70e-02 ± 1.92e-02	2.09e-02 ± 3.74e-03	1.96e-02 ± 2.33e-03
400	**3.46e-02 ± 0.00e+00**	3.12e-02 ± 3.47e-18	3.35e-02 ± 2.33e-02	2.09e-02 ± 3.74e-03	2.49e-02 ± 2.22e-03
500	3.94e-02 ± 0.00e+00	3.87e-02 ± 0.00e+00	**3.99e-02 ± 2.90e-02**	2.09e-02 ± 3.74e-03	2.92e-02 ± 2.26e-03
1000	6.09e-02 ± 6.94e-18	6.26e-02 ± 0.00e+00	**6.38e-02 ± 3.02e-02**	2.09e-02 ± 3.74e-03	4.88e-02 ± 3.73e-03
2000	9.19e-02 ± 0.00e+00	9.28e-02 ± 0.00e+00	**9.63e-02 ± 2.79e-02**	2.09e-02 ± 3.74e-03	4.90e-02 ± 3.60e-03

Table S7: Mean Top k edges Number of predictable TFs (± SD) for global GRNs against UniBind

Top K edges	ctOTVelo	OTVelo	SINCERITIES	GRNBoost2	scMTNI
100	**1.00e+00 ± 0.00e+00**	0.00e+00 ± 0.00e+00	2.00e-01 ± 4.00e-01	0.00e+00 ± 0.00e+00	2.50e-02 ± 1.56e-01
200	**2.00e+00 ± 0.00e+00**	1.00e+00 ± 0.00e+00	4.00e-01 ± 4.90e-01	0.00e+00 ± 0.00e+00	0.00e+00 ± 0.00e+00
300	2.00e+00 ± 0.00e+00	**3.00e+00 ± 0.00e+00**	4.00e-01 ± 4.90e-01	2.00e-01 ± 4.00e-01	0.00e+00 ± 0.00e+00
400	**2.00e+00 ± 0.00e+00**	**2.00e+00 ± 0.00e+00**	8.00e-01 ± 4.00e-01	0.00e+00 ± 0.00e+00	0.00e+00 ± 0.00e+00
500	**5.00e+00 ± 0.00e+00**	2.00e+00 ± 0.00e+00	8.00e-01 ± 4.00e-01	2.00e-01 ± 4.00e-01	2.50e-02 ± 1.56e-01
1000	**3.00e+00 ± 0.00e+00**	2.00e+00 ± 0.00e+00	6.00e-01 ± 8.00e-01	1.20e+00 ± 7.48e-01	0.00e+00 ± 0.00e+00
2000	**4.00e+00 ± 0.00e+00**	3.00e+00 ± 0.00e+00	1.00e+00 ± 6.32e-01	1.80e+00 ± 9.80e-01	0.00e+00 ± 0.00e+00

Table S8: Mean Top k edges Number of predictable TFs (± SD) for global GRNs against Cus_ChIP_2

Top K edges	ctOTVelo	OTVelo	SINCERITIES	GRNBoost2	scMTNI
100	**0.00e+00 ± 0.00e+00**	**0.00e+00 ± 0.00e+00**	**0.00e+00 ± 0.00e+00**	**0.00e+00 ± 0.00e+00**	**0.00e+00 ± 0.00e+00**
200	**1.00e+00 ± 0.00e+00**	0.00e+00 ± 0.00e+00	0.00e+00 ± 0.00e+00	0.00e+00 ± 0.00e+00	0.00e+00 ± 0.00e+00
300	**2.00e+00 ± 0.00e+00**	0.00e+00 ± 0.00e+00	0.00e+00 ± 0.00e+00	0.00e+00 ± 0.00e+00	0.00e+00 ± 0.00e+00
400	**1.00e+00 ± 0.00e+00**	0.00e+00 ± 0.00e+00	0.00e+00 ± 0.00e+00	0.00e+00 ± 0.00e+00	0.00e+00 ± 0.00e+00
500	**1.00e+00 ± 0.00e+00**	0.00e+00 ± 0.00e+00	0.00e+00 ± 0.00e+00	0.00e+00 ± 0.00e+00	0.00e+00 ± 0.00e+00
1000	**4.00e+00 ± 0.00e+00**	3.00e+00 ± 0.00e+00	0.00e+00 ± 0.00e+00	0.00e+00 ± 0.00e+00	0.00e+00 ± 0.00e+00
2000	**7.00e+00 ± 0.00e+00**	2.00e+00 ± 0.00e+00	4.00e-01 ± 4.90e-01	0.00e+00 ± 0.00e+00	2.50e-02 ± 1.56e-01

bioRxiv preprint doi: https://doi.org/10.64898/2026.03.11.711174; this version posted March 14, 2026. The copyright holder for this preprint (which was not certified by peer review) is the author/funder, who has granted bioRxiv a license to display the preprint in perpetuity. It is made available under aCC-BY 4.0 International license.

Table S9: Mean Top k edges Number of predictable TFs (± SD) for global GRNs against Cus_KO_0.01

Top K edges	ctOTVelo	OTVelo	SINCERITIES	GRNBoost2	scMTNI
100	0.00e+00 ± 0.00e+00	0.00e+00 ± 0.00e+00	**2.00e-01 ± 4.00e-01**	0.00e+00 ± 0.00e+00	5.00e-02 ± 3.12e-01
200	0.00e+00 ± 0.00e+00	0.00e+00 ± 0.00e+00	**2.00e-01 ± 4.00e-01**	0.00e+00 ± 0.00e+00	5.00e-02 ± 2.18e-01
300	**1.00e+00 ± 0.00e+00**	0.00e+00 ± 0.00e+00	0.00e+00 ± 0.00e+00	0.00e+00 ± 0.00e+00	2.50e-02 ± 1.56e-01
400	**1.00e+00 ± 0.00e+00**	**1.00e+00 ± 0.00e+00**	0.00e+00 ± 0.00e+00	0.00e+00 ± 0.00e+00	7.50e-02 ± 2.63e-01
500	**1.00e+00 ± 0.00e+00**	**1.00e+00 ± 0.00e+00**	2.00e-01 ± 4.00e-01	0.00e+00 ± 0.00e+00	7.50e-02 ± 2.63e-01
1000	**2.00e+00 ± 0.00e+00**	**2.00e+00 ± 0.00e+00**	2.00e-01 ± 4.00e-01	0.00e+00 ± 0.00e+00	5.00e-02 ± 2.18e-01
2000	**7.00e+00 ± 0.00e+00**	3.00e+00 ± 0.00e+00	0.00e+00 ± 0.00e+00	0.00e+00 ± 0.00e+00	5.00e-02 ± 2.18e-01

Table S10: Mean Top k edges Number of predictable TFs (± SD) for global GRNs against Cus_KO_0.01_Cus_ChIP_2_intersect

Top K edges	ctOTVelo	OTVelo	SINCERITIES	GRNBoost2	scMTNI
100	**1.00e+00 ± 0.00e+00**	0.00e+00 ± 0.00e+00	0.00e+00 ± 0.00e+00	0.00e+00 ± 0.00e+00	0.00e+00 ± 0.00e+00
200	**1.00e+00 ± 0.00e+00**	**1.00e+00 ± 0.00e+00**	0.00e+00 ± 0.00e+00	0.00e+00 ± 0.00e+00	0.00e+00 ± 0.00e+00
300	**1.00e+00 ± 0.00e+00**	**1.00e+00 ± 0.00e+00**	0.00e+00 ± 0.00e+00	0.00e+00 ± 0.00e+00	0.00e+00 ± 0.00e+00
400	**1.00e+00 ± 0.00e+00**	**1.00e+00 ± 0.00e+00**	2.00e-01 ± 4.00e-01	0.00e+00 ± 0.00e+00	0.00e+00 ± 0.00e+00
500	**1.00e+00 ± 0.00e+00**	**1.00e+00 ± 0.00e+00**	4.00e-01 ± 4.90e-01	0.00e+00 ± 0.00e+00	0.00e+00 ± 0.00e+00
1000	2.00e+00 ± 0.00e+00	**3.00e+00 ± 0.00e+00**	0.00e+00 ± 0.00e+00	0.00e+00 ± 0.00e+00	0.00e+00 ± 0.00e+00
2000	1.00e+00 ± 0.00e+00	**2.00e+00 ± 0.00e+00**	0.00e+00 ± 0.00e+00	0.00e+00 ± 0.00e+00	0.00e+00 ± 0.00e+00

Table S11: Mean Top k edges Number of predictable TFs (± SD) for global GRNs against Cus_KO_0.01_union_intersect

Top K edges	ctOTVelo	OTVelo	SINCERITIES	GRNBoost2	scMTNI
100	0.00e+00 ± 0.00e+00	0.00e+00 ± 0.00e+00	**2.00e-01 ± 4.00e-01**	0.00e+00 ± 0.00e+00	0.00e+00 ± 0.00e+00
200	0.00e+00 ± 0.00e+00	**1.00e+00 ± 0.00e+00**	0.00e+00 ± 0.00e+00	0.00e+00 ± 0.00e+00	0.00e+00 ± 0.00e+00
300	**1.00e+00 ± 0.00e+00**	0.00e+00 ± 0.00e+00	0.00e+00 ± 0.00e+00	0.00e+00 ± 0.00e+00	0.00e+00 ± 0.00e+00
400	0.00e+00 ± 0.00e+00	0.00e+00 ± 0.00e+00	0.00e+00 ± 0.00e+00	0.00e+00 ± 0.00e+00	**2.50e-02 ± 1.56e-01**
500	0.00e+00 ± 0.00e+00	0.00e+00 ± 0.00e+00	**2.00e-01 ± 4.00e-01**	0.00e+00 ± 0.00e+00	2.50e-02 ± 1.56e-01
1000	**1.00e+00 ± 0.00e+00**	**1.00e+00 ± 0.00e+00**	0.00e+00 ± 0.00e+00	0.00e+00 ± 0.00e+00	2.50e-02 ± 1.56e-01
2000	0.00e+00 ± 0.00e+00	**2.00e+00 ± 0.00e+00**	0.00e+00 ± 0.00e+00	0.00e+00 ± 0.00e+00	2.50e-02 ± 1.56e-01

Table S12: Mean Top k edges F1 score (± SD) for Hematopoietic stem cells GRNs against CD34_hematopoietic_stem_cells_CD34CD133_hematopoietic_progenitors

Top K edges	ctOTVelo	OTVelo	SINCERITIES	GRNBoost2	scMTNI
100	3.80e-03 ± 0.00e+00	3.96e-03 ± 0.00e+00	**4.29e-03 ± 1.53e-03**	3.94e-03 ± 4.65e-04	4.24e-03 ± 9.72e-04
200	7.12e-03 ± 8.67e-19	6.97e-03 ± 8.67e-19	**7.73e-03 ± 3.54e-03**	7.67e-03 ± 4.48e-04	7.41e-03 ± 3.22e-03
300	1.06e-02 ± 0.00e+00	1.02e-02 ± 0.00e+00	**1.12e-02 ± 5.11e-03**	1.12e-02 ± 5.35e-04	8.09e-03 ± 3.77e-03
400	1.46e-02 ± 1.73e-18	1.38e-02 ± 0.00e+00	**1.50e-02 ± 6.55e-03**	1.48e-02 ± 8.04e-04	8.09e-03 ± 3.77e-03
500	1.82e-02 ± 0.00e+00	1.73e-02 ± 0.00e+00	1.88e-02 ± 8.26e-03	**1.92e-02 ± 9.43e-04**	8.09e-03 ± 3.77e-03
1000	3.47e-02 ± 0.00e+00	3.24e-02 ± 0.00e+00	**3.81e-02 ± 1.57e-02**	2.09e-02 ± 1.40e-03	8.09e-03 ± 3.77e-03
2000	7.01e-02 ± 0.00e+00	6.19e-02 ± 0.00e+00	**7.83e-02 ± 2.23e-02**	2.09e-02 ± 1.40e-03	8.09e-03 ± 3.77e-03

Table S13: Mean Top k edges F1 score (± SD) for Hematopoietic stem cells GRNs against CD34_hematopoietic_stem_cells-derived_proerythroblasts

Top K edges	ctOTVelo	OTVelo	SINCERITIES	GRNBoost2	scMTNI
100	**2.67e-02 ± 0.00e+00**	**2.67e-02 ± 0.00e+00**	1.70e-02 ± 1.08e-02	2.37e-02 ± 5.98e-04	7.21e-03 ± 4.49e-03
200	**5.35e-02 ± 6.94e-18**	5.32e-02 ± 0.00e+00	3.43e-02 ± 2.14e-02	4.47e-02 ± 1.40e-03	9.55e-03 ± 8.86e-03
300	**7.84e-02 ± 0.00e+00**	7.81e-02 ± 0.00e+00	5.13e-02 ± 3.01e-02	6.55e-02 ± 1.97e-03	1.08e-02 ± 1.16e-02
400	**1.03e-01 ± 0.00e+00**	1.02e-01 ± 1.39e-17	6.74e-02 ± 3.94e-02	6.91e-02 ± 4.18e-03	1.13e-02 ± 1.25e-02
500	**1.26e-01 ± 0.00e+00**	1.25e-01 ± 0.00e+00	8.24e-02 ± 4.60e-02	6.91e-02 ± 4.18e-03	1.17e-02 ± 1.33e-02
1000	**2.32e-01 ± 0.00e+00**	2.31e-01 ± 0.00e+00	1.47e-01 ± 6.50e-02	6.91e-02 ± 4.18e-03	1.18e-02 ± 1.34e-02
2000	**4.04e-01 ± 5.55e-17**	3.96e-01 ± 0.00e+00	2.11e-01 ± 7.61e-02	6.91e-02 ± 4.18e-03	1.18e-02 ± 1.34e-02

bioRxiv preprint doi: https://doi.org/10.64898/2026.03.11.711174; this version posted March 14, 2026. The copyright holder for this preprint (which was not certified by peer review) is the author/funder, who has granted bioRxiv a license to display the preprint in perpetuity. It is made available under aCC-BY 4.0 International license.

Table S14: Mean Top k edges F1 score (± SD) for Monocyte GRNs against CD14_monocytes

Top K edges	ctOTVelo	OTVelo	SINCERITIES	GRNBoost2	scMTNI
100	**3.03e-02 ± 0.00e+00**	**3.03e-02 ± 0.00e+00**	**3.03e-02 ± 0.00e+00**	9.98e-03 ± 2.02e-03	6.64e-03 ± 1.33e-02
200	**5.97e-02 ± 0.00e+00**	**5.97e-02 ± 0.00e+00**	**5.97e-02 ± 0.00e+00**	9.98e-03 ± 2.02e-03	1.31e-02 ± 2.61e-02
300	**8.82e-02 ± 0.00e+00**	**8.82e-02 ± 0.00e+00**	**8.82e-02 ± 0.00e+00**	9.98e-03 ± 2.02e-03	1.93e-02 ± 3.86e-02
400	**1.16e-01 ± 0.00e+00**	**1.16e-01 ± 0.00e+00**	**1.16e-01 ± 0.00e+00**	9.98e-03 ± 2.02e-03	2.53e-02 ± 5.06e-02
500	**1.43e-01 ± 0.00e+00**	**1.43e-01 ± 0.00e+00**	**1.43e-01 ± 0.00e+00**	9.98e-03 ± 2.02e-03	3.11e-02 ± 6.23e-02
1000	**2.67e-01 ± 0.00e+00**	**2.67e-01 ± 0.00e+00**	2.50e-01 ± 2.93e-02	9.98e-03 ± 2.02e-03	4.97e-02 ± 9.95e-02
2000	**4.71e-01 ± 5.55e-17**	**4.71e-01 ± 5.55e-17**	3.41e-01 ± 1.13e-01	9.98e-03 ± 2.02e-03	4.97e-02 ± 9.95e-02

Table S15: Mean Top k edges F1 score (± SD) for Common myeloid progenitor GRNs against megakaryocytes

Top K edges	ctOTVelo	OTVelo	SINCERITIES	GRNBoost2	scMTNI
100	4.38e-03 ± 1.11e-03	5.42e-03 ± 0.00e+00	5.90e-03 ± 3.11e-03	**6.50e-03 ± 2.57e-04**	6.03e-03 ± 1.74e-03
200	8.25e-03 ± 9.91e-04	8.47e-03 ± 0.00e+00	**1.21e-02 ± 6.75e-03**	1.19e-02 ± 5.98e-04	1.10e-02 ± 4.57e-03
300	1.22e-02 ± 3.93e-04	1.14e-02 ± 0.00e+00	**1.87e-02 ± 9.75e-03**	1.62e-02 ± 4.83e-04	1.57e-02 ± 7.38e-03
400	1.61e-02 ± 8.28e-04	1.46e-02 ± 1.73e-18	**2.53e-02 ± 1.25e-02**	2.01e-02 ± 2.31e-04	1.78e-02 ± 8.39e-03
500	2.03e-02 ± 1.55e-03	1.82e-02 ± 0.00e+00	**3.25e-02 ± 1.48e-02**	2.46e-02 ± 7.20e-04	1.84e-02 ± 8.77e-03
1000	4.19e-02 ± 6.17e-03	3.41e-02 ± 0.00e+00	**6.81e-02 ± 2.65e-02**	3.48e-02 ± 1.59e-03	1.84e-02 ± 8.77e-03
2000	8.59e-02 ± 1.85e-02	6.08e-02 ± 6.94e-18	**1.25e-01 ± 4.73e-02**	3.48e-02 ± 1.59e-03	1.84e-02 ± 8.77e-03

Table S16: Mean Top k edges F1 score (± SD) for Common myeloid progenitor GRNs against erythroid_progenitors

Top K edges	ctOTVelo	OTVelo	SINCERITIES	GRNBoost2	scMTNI
100	**2.49e-02 ± 6.94e-18**	2.49e-02 ± 0.00e+00	2.49e-02 ± 0.00e+00	9.54e-03 ± 1.67e-03	2.21e-02 ± 1.03e-02
200	**4.92e-02 ± 1.39e-17**	4.92e-02 ± 0.00e+00	4.92e-02 ± 0.00e+00	9.54e-03 ± 1.67e-03	2.42e-02 ± 1.22e-02
300	7.29e-02 ± 1.39e-17	**7.29e-02 ± 0.00e+00**	**7.29e-02 ± 0.00e+00**	9.54e-03 ± 1.67e-03	2.42e-02 ± 1.22e-02
400	**9.61e-02 ± 1.39e-17**	9.61e-02 ± 0.00e+00	9.61e-02 ± 0.00e+00	9.54e-03 ± 1.67e-03	2.42e-02 ± 1.22e-02
500	1.19e-01 ± 0.00e+00	**1.19e-01 ± 1.39e-17**	**1.19e-01 ± 1.39e-17**	9.54e-03 ± 1.67e-03	2.42e-02 ± 1.22e-02
1000	2.24e-01 ± 2.78e-17	**2.24e-01 ± 0.00e+00**	2.18e-01 ± 1.17e-02	9.54e-03 ± 1.67e-03	2.42e-02 ± 1.22e-02
2000	4.03e-01 ± 0.00e+00	**4.03e-01 ± 5.55e-17**	3.13e-01 ± 8.18e-02	9.54e-03 ± 1.67e-03	2.42e-02 ± 1.22e-02

Table S17: Mean Top k edges F1 score (± SD) for Common myeloid progenitor GRNs against B-cells

Top K edges	ctOTVelo	OTVelo	SINCERITIES	GRNBoost2	scMTNI
100	**2.81e-02 ± 6.94e-18**	2.81e-02 ± 0.00e+00	2.81e-02 ± 0.00e+00	9.59e-03 ± 1.74e-03	2.31e-02 ± 1.09e-02
200	**5.55e-02 ± 1.39e-17**	5.55e-02 ± 0.00e+00	5.55e-02 ± 0.00e+00	9.59e-03 ± 1.74e-03	2.42e-02 ± 1.22e-02
300	**8.20e-02 ± 2.78e-17**	8.20e-02 ± 0.00e+00	8.20e-02 ± 0.00e+00	9.59e-03 ± 1.74e-03	2.42e-02 ± 1.22e-02
400	**1.08e-01 ± 4.16e-17**	1.08e-01 ± 1.39e-17	1.08e-01 ± 1.39e-17	9.59e-03 ± 1.74e-03	2.42e-02 ± 1.22e-02
500	**1.33e-01 ± 0.00e+00**	**1.33e-01 ± 0.00e+00**	**1.33e-01 ± 0.00e+00**	9.59e-03 ± 1.74e-03	2.42e-02 ± 1.22e-02
1000	**2.50e-01 ± 2.78e-17**	2.50e-01 ± 0.00e+00	2.38e-01 ± 2.25e-02	9.59e-03 ± 1.74e-03	2.42e-02 ± 1.22e-02
2000	4.44e-01 ± 0.00e+00	**4.44e-01 ± 5.55e-17**	3.30e-01 ± 1.01e-01	9.59e-03 ± 1.74e-03	2.42e-02 ± 1.22e-02

Table S18: Mean Top k edges F1 score (± SD) for Common myeloid progenitor GRNs against R3R4_erythroid_cells

Top K edges	ctOTVelo	OTVelo	SINCERITIES	GRNBoost2	scMTNI
100	**2.33e-02 ± 0.00e+00**	**2.33e-02 ± 0.00e+00**	**2.33e-02 ± 0.00e+00**	1.81e-02 ± 9.90e-04	2.04e-02 ± 1.03e-02
200	**4.61e-02 ± 6.94e-18**	4.61e-02 ± 0.00e+00	4.61e-02 ± 0.00e+00	1.81e-02 ± 9.90e-04	2.48e-02 ± 1.44e-02
300	6.84e-02 ± 2.78e-17	**6.84e-02 ± 0.00e+00**	**6.84e-02 ± 0.00e+00**	1.81e-02 ± 9.90e-04	2.48e-02 ± 1.44e-02
400	**9.02e-02 ± 2.78e-17**	9.02e-02 ± 0.00e+00	9.02e-02 ± 0.00e+00	1.81e-02 ± 9.90e-04	2.48e-02 ± 1.44e-02
500	**1.11e-01 ± 0.00e+00**	**1.11e-01 ± 0.00e+00**	**1.11e-01 ± 0.00e+00**	1.81e-02 ± 9.90e-04	2.48e-02 ± 1.44e-02
1000	**2.11e-01 ± 8.33e-17**	2.11e-01 ± 2.78e-17	1.89e-01 ± 2.99e-02	1.81e-02 ± 9.90e-04	2.48e-02 ± 1.44e-02
2000	3.82e-01 ± 1.11e-16	**3.82e-01 ± 0.00e+00**	2.92e-01 ± 1.11e-01	1.81e-02 ± 9.90e-04	2.48e-02 ± 1.44e-02

bioRxiv preprint doi: https://doi.org/10.64898/2026.03.11.711174; this version posted March 14, 2026. The copyright holder for this preprint (which was not certified by peer review) is the author/funder, who has granted bioRxiv a license to display the preprint in perpetuity. It is made available under aCC-BY 4.0 International license.

Table S19: Mean Top k edges F1 score (± SD) for Granulocyte-marophage progenitors GRNs against GM_B-cells

Top K edges	ctOTVelo	OTVelo	SINCERITIES	GRNBoost2	scMTNI
100	2.59e-04 ± 0.00e+00	3.73e-04 ± 0.00e+00	**4.97e-04 ± 2.67e-04**	3.48e-04 ± 4.37e-05	2.75e-04 ± 1.46e-04
200	6.31e-04 ± 0.00e+00	8.80e-04 ± 1.08e-19	**1.00e-03 ± 4.94e-04**	7.02e-04 ± 8.94e-05	6.11e-04 ± 2.64e-04
300	1.12e-03 ± 0.00e+00	1.43e-03 ± 0.00e+00	**1.46e-03 ± 6.91e-04**	1.04e-03 ± 8.10e-05	1.02e-03 ± 3.10e-04
400	1.58e-03 ± 2.17e-19	**2.09e-03 ± 0.00e+00**	1.84e-03 ± 8.44e-04	1.45e-03 ± 6.69e-05	1.43e-03 ± 3.40e-04
500	2.07e-03 ± 0.00e+00	**2.62e-03 ± 0.00e+00**	2.20e-03 ± 9.77e-04	1.80e-03 ± 5.97e-05	1.82e-03 ± 3.93e-04
1000	4.38e-03 ± 0.00e+00	**5.45e-03 ± 0.00e+00**	4.17e-03 ± 1.65e-03	3.72e-03 ± 1.08e-04	3.97e-03 ± 5.54e-04
2000	9.50e-03 ± 0.00e+00	**1.10e-02 ± 0.00e+00**	9.06e-03 ± 2.28e-03	7.63e-03 ± 1.56e-04	7.12e-03 ± 2.05e-03

Table S20: Mean Top k edges F1 score (± SD) for CD34 GRNs against CD34_hematopoietic_stem_cells_CD34CD133_hematopoietic_progenitors

Top K edges	ctOTVelo	OTVelo	SINCERITIES	GRNBoost2	scMTNI
100	3.96e-03 ± 0.00e+00	3.96e-03 ± 0.00e+00	**4.29e-03 ± 1.53e-03**	3.94e-03 ± 4.65e-04	4.24e-03 ± 9.72e-04
200	7.66e-03 ± 0.00e+00	6.97e-03 ± 8.67e-19	**7.73e-03 ± 3.54e-03**	7.67e-03 ± 4.48e-04	7.41e-03 ± 3.22e-03
300	1.06e-02 ± 0.00e+00	1.02e-02 ± 0.00e+00	**1.12e-02 ± 5.11e-03**	1.12e-02 ± 5.35e-04	8.09e-03 ± 3.77e-03
400	1.36e-02 ± 0.00e+00	1.38e-02 ± 0.00e+00	**1.50e-02 ± 6.55e-03**	1.48e-02 ± 8.04e-04	8.09e-03 ± 3.77e-03
500	1.69e-02 ± 0.00e+00	1.73e-02 ± 0.00e+00	1.88e-02 ± 8.26e-03	**1.92e-02 ± 9.43e-04**	8.09e-03 ± 3.77e-03
1000	3.13e-02 ± 0.00e+00	3.24e-02 ± 0.00e+00	**3.81e-02 ± 1.57e-02**	2.09e-02 ± 1.40e-03	8.09e-03 ± 3.77e-03
2000	5.74e-02 ± 0.00e+00	6.19e-02 ± 0.00e+00	**7.83e-02 ± 2.23e-02**	2.09e-02 ± 1.40e-03	8.09e-03 ± 3.77e-03

Table S21: Mean Top k edges F1 score (± SD) for CD34 GRNs against CD34_hematopoietic_stem_cells-derived_proerythroblasts

Top K edges	ctOTVelo	OTVelo	SINCERITIES	GRNBoost2	scMTNI
100	**2.76e-02 ± 3.47e-18**	2.67e-02 ± 0.00e+00	1.70e-02 ± 1.08e-02	2.37e-02 ± 5.98e-04	7.21e-03 ± 4.49e-03
200	**5.38e-02 ± 0.00e+00**	5.32e-02 ± 0.00e+00	3.43e-02 ± 2.14e-02	4.47e-02 ± 1.40e-03	9.55e-03 ± 8.86e-03
300	**7.81e-02 ± 0.00e+00**	**7.81e-02 ± 0.00e+00**	5.13e-02 ± 3.01e-02	6.55e-02 ± 1.97e-03	1.08e-02 ± 1.16e-02
400	**1.02e-01 ± 1.39e-17**	1.02e-01 ± 1.39e-17	6.74e-02 ± 3.94e-02	6.91e-02 ± 4.18e-03	1.13e-02 ± 1.25e-02
500	**1.26e-01 ± 0.00e+00**	1.25e-01 ± 0.00e+00	8.24e-02 ± 4.60e-02	6.91e-02 ± 4.18e-03	1.17e-02 ± 1.33e-02
1000	2.30e-01 ± 0.00e+00	**2.31e-01 ± 0.00e+00**	1.47e-01 ± 6.50e-02	6.91e-02 ± 4.18e-03	1.18e-02 ± 1.34e-02
2000	**4.00e-01 ± 0.00e+00**	3.96e-01 ± 0.00e+00	2.11e-01 ± 7.61e-02	6.91e-02 ± 4.18e-03	1.18e-02 ± 1.34e-02

Table S22: Mean Top k edges Number of predictable TFs (± SD) for Hematopoietic stem cells GRNs against CD34_hematopoietic_stem_cells_CD34CD133_hematopoietic_progenitors

Top K edges	ctOTVelo	OTVelo	SINCERITIES	GRNBoost2	scMTNI
100	**1.00e+00 ± 0.00e+00**	**1.00e+00 ± 0.00e+00**	0.00e+00 ± 0.00e+00	6.00e-01 ± 4.90e-01	0.00e+00 ± 0.00e+00
200	**1.00e+00 ± 0.00e+00**	**1.00e+00 ± 0.00e+00**	0.00e+00 ± 0.00e+00	**1.00e+00 ± 0.00e+00**	6.67e-02 ± 2.49e-01
300	0.00e+00 ± 0.00e+00	**1.00e+00 ± 0.00e+00**	2.00e-01 ± 4.00e-01	8.00e-01 ± 4.00e-01	6.67e-02 ± 2.49e-01
400	0.00e+00 ± 0.00e+00	**1.00e+00 ± 0.00e+00**	2.00e-01 ± 4.00e-01	8.00e-01 ± 7.48e-01	6.67e-02 ± 2.49e-01
500	0.00e+00 ± 0.00e+00	**1.00e+00 ± 0.00e+00**	0.00e+00 ± 0.00e+00	8.00e-01 ± 7.48e-01	6.67e-02 ± 2.49e-01
1000	0.00e+00 ± 0.00e+00	**1.00e+00 ± 0.00e+00**	4.00e-01 ± 4.90e-01	8.00e-01 ± 7.48e-01	6.67e-02 ± 2.49e-01
2000	0.00e+00 ± 0.00e+00	**1.00e+00 ± 0.00e+00**	2.00e-01 ± 4.00e-01	8.00e-01 ± 7.48e-01	6.67e-02 ± 2.49e-01

Table S23: Mean Top k edges Number of predictable TFs (± SD) for Hematopoietic stem cells GRNs against CD34_hematopoietic_stem_cells-derived_proerythroblasts

Top K edges	ctOTVelo	OTVelo	SINCERITIES	GRNBoost2	scMTNI
100	0.00e+00 ± 0.00e+00	0.00e+00 ± 0.00e+00	0.00e+00 ± 0.00e+00	**6.00e-01 ± 4.90e-01**	0.00e+00 ± 0.00e+00
200	**1.00e+00 ± 0.00e+00**	**1.00e+00 ± 0.00e+00**	0.00e+00 ± 0.00e+00	6.00e-01 ± 4.90e-01	0.00e+00 ± 0.00e+00
300	**1.00e+00 ± 0.00e+00**	**1.00e+00 ± 0.00e+00**	0.00e+00 ± 0.00e+00	4.00e-01 ± 4.90e-01	0.00e+00 ± 0.00e+00
400	**1.00e+00 ± 0.00e+00**	0.00e+00 ± 0.00e+00	0.00e+00 ± 0.00e+00	4.00e-01 ± 4.90e-01	0.00e+00 ± 0.00e+00
500	**1.00e+00 ± 0.00e+00**	0.00e+00 ± 0.00e+00	0.00e+00 ± 0.00e+00	4.00e-01 ± 4.90e-01	0.00e+00 ± 0.00e+00
1000	0.00e+00 ± 0.00e+00	0.00e+00 ± 0.00e+00	0.00e+00 ± 0.00e+00	**4.00e-01 ± 4.90e-01**	0.00e+00 ± 0.00e+00
2000	0.00e+00 ± 0.00e+00	0.00e+00 ± 0.00e+00	0.00e+00 ± 0.00e+00	**4.00e-01 ± 4.90e-01**	0.00e+00 ± 0.00e+00

bioRxiv preprint doi: https://doi.org/10.64898/2026.03.11.711174; this version posted March 14, 2026. The copyright holder for this preprint (which was not certified by peer review) is the author/funder, who has granted bioRxiv a license to display the preprint in perpetuity. It is made available under aCC-BY 4.0 International license.

Table S24: Mean Top k edges Number of predictable TFs (± SD) for Monocyte GRNs against CD14_monocytes

Top K edges	ctOTVelo	OTVelo	SINCERITIES	GRNBoost2	scMTNI
100	**0.00e+00 ± 0.00e+00**	**0.00e+00 ± 0.00e+00**	**0.00e+00 ± 0.00e+00**	**0.00e+00 ± 0.00e+00**	**0.00e+00 ± 0.00e+00**
200	**0.00e+00 ± 0.00e+00**	**0.00e+00 ± 0.00e+00**	**0.00e+00 ± 0.00e+00**	**0.00e+00 ± 0.00e+00**	**0.00e+00 ± 0.00e+00**
300	**0.00e+00 ± 0.00e+00**	**0.00e+00 ± 0.00e+00**	**0.00e+00 ± 0.00e+00**	**0.00e+00 ± 0.00e+00**	**0.00e+00 ± 0.00e+00**
400	**0.00e+00 ± 0.00e+00**	**0.00e+00 ± 0.00e+00**	**0.00e+00 ± 0.00e+00**	**0.00e+00 ± 0.00e+00**	**0.00e+00 ± 0.00e+00**
500	**0.00e+00 ± 0.00e+00**	**0.00e+00 ± 0.00e+00**	**0.00e+00 ± 0.00e+00**	**0.00e+00 ± 0.00e+00**	**0.00e+00 ± 0.00e+00**
1000	**0.00e+00 ± 0.00e+00**	**0.00e+00 ± 0.00e+00**	**0.00e+00 ± 0.00e+00**	**0.00e+00 ± 0.00e+00**	**0.00e+00 ± 0.00e+00**
2000	**0.00e+00 ± 0.00e+00**	**0.00e+00 ± 0.00e+00**	**0.00e+00 ± 0.00e+00**	**0.00e+00 ± 0.00e+00**	**0.00e+00 ± 0.00e+00**

Table S25: Mean Top k edges Number of predictable TFs (± SD) for Common myeloid progenitors GRNs against megakaryocytes

Top K edges	ctOTVelo	OTVelo	SINCERITIES	GRNBoost2	scMTNI
100	0.00e+00 ± 0.00e+00	**2.00e+00 ± 0.00e+00**	0.00e+00 ± 0.00e+00	1.80e+00 ± 4.00e-01	5.00e-02 ± 2.18e-01
200	0.00e+00 ± 0.00e+00	2.00e+00 ± 0.00e+00	0.00e+00 ± 0.00e+00	**2.20e+00 ± 4.00e-01**	5.00e-02 ± 2.18e-01
300	0.00e+00 ± 0.00e+00	**2.00e+00 ± 0.00e+00**	0.00e+00 ± 0.00e+00	**2.00e+00 ± 0.00e+00**	5.00e-02 ± 2.18e-01
400	0.00e+00 ± 0.00e+00	**2.00e+00 ± 0.00e+00**	2.00e-01 ± 4.00e-01	**2.00e+00 ± 0.00e+00**	5.00e-02 ± 2.18e-01
500	0.00e+00 ± 0.00e+00	**2.00e+00 ± 0.00e+00**	2.00e-01 ± 4.00e-01	**2.00e+00 ± 0.00e+00**	5.00e-02 ± 2.18e-01
1000	0.00e+00 ± 0.00e+00	**2.00e+00 ± 0.00e+00**	4.00e-01 ± 4.90e-01	1.80e+00 ± 4.00e-01	5.00e-02 ± 2.18e-01
2000	**3.00e+00 ± 0.00e+00**	1.00e+00 ± 0.00e+00	4.00e-01 ± 4.90e-01	1.80e+00 ± 4.00e-01	5.00e-02 ± 2.18e-01

Table S26: Mean Top k edges Number of predictable TFs (± SD) for Common myeloid progenitors GRNs against erythroid_progenitors

Top K edges	ctOTVelo	OTVelo	SINCERITIES	GRNBoost2	scMTNI
100	**0.00e+00 ± 0.00e+00**	**0.00e+00 ± 0.00e+00**	**0.00e+00 ± 0.00e+00**	**0.00e+00 ± 0.00e+00**	**0.00e+00 ± 0.00e+00**
200	**0.00e+00 ± 0.00e+00**	**0.00e+00 ± 0.00e+00**	**0.00e+00 ± 0.00e+00**	**0.00e+00 ± 0.00e+00**	**0.00e+00 ± 0.00e+00**
300	**0.00e+00 ± 0.00e+00**	**0.00e+00 ± 0.00e+00**	**0.00e+00 ± 0.00e+00**	**0.00e+00 ± 0.00e+00**	**0.00e+00 ± 0.00e+00**
400	**0.00e+00 ± 0.00e+00**	**0.00e+00 ± 0.00e+00**	**0.00e+00 ± 0.00e+00**	**0.00e+00 ± 0.00e+00**	**0.00e+00 ± 0.00e+00**
500	**0.00e+00 ± 0.00e+00**	**0.00e+00 ± 0.00e+00**	**0.00e+00 ± 0.00e+00**	**0.00e+00 ± 0.00e+00**	**0.00e+00 ± 0.00e+00**
1000	**0.00e+00 ± 0.00e+00**	**0.00e+00 ± 0.00e+00**	**0.00e+00 ± 0.00e+00**	**0.00e+00 ± 0.00e+00**	**0.00e+00 ± 0.00e+00**
2000	**0.00e+00 ± 0.00e+00**	**0.00e+00 ± 0.00e+00**	**0.00e+00 ± 0.00e+00**	**0.00e+00 ± 0.00e+00**	**0.00e+00 ± 0.00e+00**

Table S27: Mean Top k edges Number of predictable TFs (± SD) for Common myeloid progenitors GRNs against B-cells

Top K edges	ctOTVelo	OTVelo	SINCERITIES	GRNBoost2	scMTNI
100	**0.00e+00 ± 0.00e+00**	**0.00e+00 ± 0.00e+00**	**0.00e+00 ± 0.00e+00**	**0.00e+00 ± 0.00e+00**	**0.00e+00 ± 0.00e+00**
200	**0.00e+00 ± 0.00e+00**	**0.00e+00 ± 0.00e+00**	**0.00e+00 ± 0.00e+00**	**0.00e+00 ± 0.00e+00**	**0.00e+00 ± 0.00e+00**
300	**0.00e+00 ± 0.00e+00**	**0.00e+00 ± 0.00e+00**	**0.00e+00 ± 0.00e+00**	**0.00e+00 ± 0.00e+00**	**0.00e+00 ± 0.00e+00**
400	**0.00e+00 ± 0.00e+00**	**0.00e+00 ± 0.00e+00**	**0.00e+00 ± 0.00e+00**	**0.00e+00 ± 0.00e+00**	**0.00e+00 ± 0.00e+00**
500	**0.00e+00 ± 0.00e+00**	**0.00e+00 ± 0.00e+00**	**0.00e+00 ± 0.00e+00**	**0.00e+00 ± 0.00e+00**	**0.00e+00 ± 0.00e+00**
1000	**0.00e+00 ± 0.00e+00**	**0.00e+00 ± 0.00e+00**	**0.00e+00 ± 0.00e+00**	**0.00e+00 ± 0.00e+00**	**0.00e+00 ± 0.00e+00**
2000	**0.00e+00 ± 0.00e+00**	**0.00e+00 ± 0.00e+00**	**0.00e+00 ± 0.00e+00**	**0.00e+00 ± 0.00e+00**	**0.00e+00 ± 0.00e+00**

Table S28: Mean Top k edges Number of predictable TFs (± SD) for Common myeloid progenitors GRNs against R3R4_erythroid_cells

Top K edges	ctOTVelo	OTVelo	SINCERITIES	GRNBoost2	scMTNI
100	**0.00e+00 ± 0.00e+00**	**0.00e+00 ± 0.00e+00**	**0.00e+00 ± 0.00e+00**	**0.00e+00 ± 0.00e+00**	**0.00e+00 ± 0.00e+00**
200	**0.00e+00 ± 0.00e+00**	**0.00e+00 ± 0.00e+00**	**0.00e+00 ± 0.00e+00**	**0.00e+00 ± 0.00e+00**	**0.00e+00 ± 0.00e+00**
300	**0.00e+00 ± 0.00e+00**	**0.00e+00 ± 0.00e+00**	**0.00e+00 ± 0.00e+00**	**0.00e+00 ± 0.00e+00**	**0.00e+00 ± 0.00e+00**
400	**0.00e+00 ± 0.00e+00**	**0.00e+00 ± 0.00e+00**	**0.00e+00 ± 0.00e+00**	**0.00e+00 ± 0.00e+00**	**0.00e+00 ± 0.00e+00**
500	**0.00e+00 ± 0.00e+00**	**0.00e+00 ± 0.00e+00**	**0.00e+00 ± 0.00e+00**	**0.00e+00 ± 0.00e+00**	**0.00e+00 ± 0.00e+00**
1000	**0.00e+00 ± 0.00e+00**	**0.00e+00 ± 0.00e+00**	**0.00e+00 ± 0.00e+00**	**0.00e+00 ± 0.00e+00**	**0.00e+00 ± 0.00e+00**
2000	**0.00e+00 ± 0.00e+00**	**0.00e+00 ± 0.00e+00**	**0.00e+00 ± 0.00e+00**	**0.00e+00 ± 0.00e+00**	**0.00e+00 ± 0.00e+00**

bioRxiv preprint doi: https://doi.org/10.64898/2026.03.11.711174; this version posted March 14, 2026. The copyright holder for this preprint (which was not certified by peer review) is the author/funder, who has granted bioRxiv a license to display the preprint in perpetuity. It is made available under aCC-BY 4.0 International license.

Table S29: Mean Top k edges Number of predictable TFs (± SD) for Granulocyte-macrophage progenitors GRNs against GM_B-cells

Top K edges	ctOTVelo	OTVelo	SINCERITIES	GRNBoost2	scMTNI
100	**0.00e+00 ± 0.00e+00**	**0.00e+00 ± 0.00e+00**	**0.00e+00 ± 0.00e+00**	**0.00e+00 ± 0.00e+00**	**0.00e+00 ± 0.00e+00**
200	0.00e+00 ± 0.00e+00	**1.00e+00 ± 0.00e+00**	4.00e-01 ± 4.90e-01	0.00e+00 ± 0.00e+00	0.00e+00 ± 0.00e+00
300	0.00e+00 ± 0.00e+00	**1.00e+00 ± 0.00e+00**	2.00e-01 ± 4.00e-01	0.00e+00 ± 0.00e+00	0.00e+00 ± 0.00e+00
400	0.00e+00 ± 0.00e+00	**2.00e+00 ± 0.00e+00**	4.00e-01 ± 4.90e-01	0.00e+00 ± 0.00e+00	0.00e+00 ± 0.00e+00
500	0.00e+00 ± 0.00e+00	0.00e+00 ± 0.00e+00	**4.00e-01 ± 4.90e-01**	0.00e+00 ± 0.00e+00	0.00e+00 ± 0.00e+00
1000	0.00e+00 ± 0.00e+00	**2.00e+00 ± 0.00e+00**	8.00e-01 ± 1.17e+00	0.00e+00 ± 0.00e+00	0.00e+00 ± 0.00e+00
2000	0.00e+00 ± 0.00e+00	1.00e+00 ± 0.00e+00	**1.40e+00 ± 1.02e+00**	4.00e-01 ± 8.00e-01	0.00e+00 ± 0.00e+00

Table S30: Mean Top k edges Number of predictable TFs (± SD) for CD34 GRNs against CD34_hematopoietic_stem_cells_CD34CD133_hematopoietic_progenitors

Top K edges	ctOTVelo	OTVelo	SINCERITIES	GRNBoost2	scMTNI
100	**1.00e+00 ± 0.00e+00**	**1.00e+00 ± 0.00e+00**	0.00e+00 ± 0.00e+00	6.00e-01 ± 4.90e-01	0.00e+00 ± 0.00e+00
200	**1.00e+00 ± 0.00e+00**	**1.00e+00 ± 0.00e+00**	0.00e+00 ± 0.00e+00	**1.00e+00 ± 0.00e+00**	6.67e-02 ± 2.49e-01
300	**1.00e+00 ± 0.00e+00**	**1.00e+00 ± 0.00e+00**	2.00e-01 ± 4.00e-01	8.00e-01 ± 4.00e-01	6.67e-02 ± 2.49e-01
400	**1.00e+00 ± 0.00e+00**	**1.00e+00 ± 0.00e+00**	2.00e-01 ± 4.00e-01	8.00e-01 ± 7.48e-01	6.67e-02 ± 2.49e-01
500	**1.00e+00 ± 0.00e+00**	**1.00e+00 ± 0.00e+00**	0.00e+00 ± 0.00e+00	8.00e-01 ± 7.48e-01	6.67e-02 ± 2.49e-01
1000	**1.00e+00 ± 0.00e+00**	**1.00e+00 ± 0.00e+00**	4.00e-01 ± 4.90e-01	8.00e-01 ± 7.48e-01	6.67e-02 ± 2.49e-01
2000	**1.00e+00 ± 0.00e+00**	**1.00e+00 ± 0.00e+00**	2.00e-01 ± 4.00e-01	8.00e-01 ± 7.48e-01	6.67e-02 ± 2.49e-01

Table S31: Mean Top k edges Number of predictable TFs (± SD) for CD34 GRNs against CD34_hematopoietic_stem_cells-derived_proerythroblasts

Top K edges	ctOTVelo	OTVelo	SINCERITIES	GRNBoost2	scMTNI
100	**1.00e+00 ± 0.00e+00**	0.00e+00 ± 0.00e+00	0.00e+00 ± 0.00e+00	6.00e-01 ± 4.90e-01	0.00e+00 ± 0.00e+00
200	**1.00e+00 ± 0.00e+00**	**1.00e+00 ± 0.00e+00**	0.00e+00 ± 0.00e+00	6.00e-01 ± 4.90e-01	0.00e+00 ± 0.00e+00
300	**1.00e+00 ± 0.00e+00**	**1.00e+00 ± 0.00e+00**	0.00e+00 ± 0.00e+00	4.00e-01 ± 4.90e-01	0.00e+00 ± 0.00e+00
400	**1.00e+00 ± 0.00e+00**	0.00e+00 ± 0.00e+00	0.00e+00 ± 0.00e+00	4.00e-01 ± 4.90e-01	0.00e+00 ± 0.00e+00
500	**1.00e+00 ± 0.00e+00**	0.00e+00 ± 0.00e+00	0.00e+00 ± 0.00e+00	4.00e-01 ± 4.90e-01	0.00e+00 ± 0.00e+00
1000	0.00e+00 ± 0.00e+00	0.00e+00 ± 0.00e+00	0.00e+00 ± 0.00e+00	**4.00e-01 ± 4.90e-01**	0.00e+00 ± 0.00e+00
2000	0.00e+00 ± 0.00e+00	0.00e+00 ± 0.00e+00	0.00e+00 ± 0.00e+00	**4.00e-01 ± 4.90e-01**	0.00e+00 ± 0.00e+00

C Full quantitative metrics for mouse organogenesis

Table S32: Mean Top k edges F1 score (± SD) for global GRNs against mESC_chipunion

Top K edges	ctOTVelo	OTVelo	SINCERITIES	GRNBoost2	scMTNI
100	**3.31e-03 ± 5.29e-05**	1.32e-03 ± 8.09e-05	1.28e-03 ± 8.24e-04	1.92e-03 ± 2.20e-04	9.06e-04 ± 1.73e-04
200	**5.03e-03 ± 4.30e-05**	2.34e-03 ± 4.30e-05	2.32e-03 ± 1.73e-03	3.27e-03 ± 3.51e-04	1.66e-03 ± 1.51e-04
300	**6.59e-03 ± 1.45e-04**	3.25e-03 ± 1.74e-04	3.85e-03 ± 2.62e-03	4.77e-03 ± 4.67e-04	2.54e-03 ± 3.28e-04
400	**8.38e-03 ± 1.70e-04**	5.21e-03 ± 1.90e-04	5.42e-03 ± 3.43e-03	6.25e-03 ± 5.48e-04	3.26e-03 ± 5.04e-04
500	**1.03e-02 ± 2.89e-04**	6.45e-03 ± 1.16e-04	7.02e-03 ± 4.25e-03	7.57e-03 ± 9.89e-04	4.00e-03 ± 4.50e-04
1000	**2.09e-02 ± 2.39e-04**	1.49e-02 ± 3.57e-04	1.42e-02 ± 7.68e-03	1.46e-02 ± 8.71e-04	7.35e-03 ± 3.28e-04
2000	**3.87e-02 ± 2.81e-04**	3.10e-02 ± 7.40e-04	2.77e-02 ± 1.10e-02	2.36e-02 ± 9.10e-04	1.37e-02 ± 3.93e-04

Table S33: Mean Top k edges F1 score (± SD) for global GRNs against mESC_KDUnion

Top K edges	ctOTVelo	OTVelo	SINCERITIES	GRNBoost2	scMTNI
100	2.46e-04 ± 1.70e-05	1.61e-04 ± 1.70e-05	2.63e-04 ± 3.24e-04	**3.73e-04 ± 9.82e-05**	2.03e-04 ± 6.45e-05
200	3.47e-04 ± 6.77e-05	2.79e-04 ± 3.39e-05	5.08e-04 ± 5.91e-04	**8.89e-04 ± 5.99e-05**	4.47e-04 ± 1.07e-04
300	6.08e-04 ± 7.84e-05	4.31e-04 ± 1.69e-05	8.11e-04 ± 6.51e-04	**1.34e-03 ± 1.68e-04**	6.75e-04 ± 1.18e-04
400	9.87e-04 ± 5.72e-05	5.90e-04 ± 3.77e-05	1.10e-03 ± 7.26e-04	**1.70e-03 ± 1.66e-04**	9.03e-04 ± 1.55e-04
500	1.30e-03 ± 1.17e-04	8.92e-04 ± 4.12e-05	1.50e-03 ± 8.29e-04	**2.14e-03 ± 2.08e-04**	1.15e-03 ± 1.25e-04
1000	3.29e-03 ± 1.81e-04	2.26e-03 ± 8.57e-05	2.94e-03 ± 1.16e-03	**4.00e-03 ± 3.26e-04**	2.25e-03 ± 1.10e-04
2000	**8.42e-03 ± 1.78e-04**	6.53e-03 ± 2.10e-04	5.53e-03 ± 2.29e-03	7.77e-03 ± 2.99e-04	4.39e-03 ± 1.82e-04

bioRxiv preprint doi: https://doi.org/10.64898/2026.03.11.711174; this version posted March 14, 2026. The copyright holder for this preprint (which was not certified by peer review) is the author/funder, who has granted bioRxiv a license to display the preprint in perpetuity. It is made available under aCC-BY 4.0 International license.

Table S34: Mean Top k edges F1 score (± SD) for global GRNs against mESC_chipunion_KDUnion_intersect

Top K edges	ctOTVelo	OTVelo	SINCERITIES	GRNBoost2	scMTNI
100	**1.94e-02** ± **5.11e-04**	5.49e-03 ± 3.23e-04	6.78e-03 ± 5.27e-03	1.42e-02 ± 2.08e-03	7.20e-03 ± 1.48e-03
200	**3.65e-02** ± **6.94e-04**	1.65e-02 ± 3.10e-04	1.16e-02 ± 6.82e-03	2.30e-02 ± 2.43e-03	1.26e-02 ± 2.55e-03
300	**5.11e-02** ± **7.62e-04**	2.81e-02 ± 1.21e-03	1.34e-02 ± 8.67e-03	2.86e-02 ± 3.14e-03	1.51e-02 ± 2.00e-03
400	**6.28e-02** ± **1.84e-03**	3.66e-02 ± 1.40e-03	1.67e-02 ± 7.45e-03	2.88e-02 ± 3.37e-03	1.51e-02 ± 2.00e-03
500	**7.55e-02** ± **3.28e-03**	4.48e-02 ± 8.34e-04	1.88e-02 ± 6.25e-03	2.88e-02 ± 3.37e-03	1.51e-02 ± 2.00e-03
1000	**1.30e-01** ± **1.73e-03**	9.70e-02 ± 2.52e-03	2.98e-02 ± 5.34e-03	2.88e-02 ± 3.37e-03	1.51e-02 ± 2.00e-03
2000	**1.70e-01** ± **1.60e-03**	1.46e-01 ± 2.80e-03	4.80e-02 ± 8.06e-03	2.88e-02 ± 3.37e-03	1.51e-02 ± 2.00e-03

Table S35: Mean Top k edges Precision (± SD) for global GRNs against mESC_chipunion

Top K edges	ctOTVelo	OTVelo	SINCERITIES	GRNBoost2	scMTNI
100	**3.06e-01** ± **4.90e-03**	1.22e-01 ± 7.48e-03	1.18e-01 ± 7.63e-02	1.78e-01 ± 2.04e-02	1.90e-01 ± 3.63e-02
200	**2.34e-01** ± **2.00e-03**	1.09e-01 ± 2.00e-03	1.08e-01 ± 8.05e-02	1.52e-01 ± 1.63e-02	1.74e-01 ± 1.59e-02
300	**2.05e-01** ± **4.52e-03**	1.01e-01 ± 5.42e-03	1.20e-01 ± 8.17e-02	1.49e-01 ± 1.45e-02	1.78e-01 ± 2.31e-02
400	**1.97e-01** ± **4.00e-03**	1.23e-01 ± 4.47e-03	1.28e-01 ± 8.06e-02	1.47e-01 ± 1.29e-02	1.72e-01 ± 2.67e-02
500	**1.95e-01** ± **5.46e-03**	1.22e-01 ± 2.19e-03	1.33e-01 ± 8.03e-02	1.43e-01 ± 1.87e-02	1.69e-01 ± 1.91e-02
1000	**2.03e-01** ± **2.32e-03**	1.45e-01 ± 3.46e-03	1.38e-01 ± 7.46e-02	1.42e-01 ± 8.45e-03	1.57e-01 ± 7.03e-03
2000	**1.97e-01** ± **1.44e-03**	1.58e-01 ± 3.77e-03	1.41e-01 ± 5.61e-02	1.39e-01 ± 5.39e-03	1.50e-01 ± 4.36e-03

Table S36: Mean Top k edges Precision (± SD) for global GRNs against mESC_KDUnion

Top K edges	ctOTVelo	OTVelo	SINCERITIES	GRNBoost2	scMTNI
100	5.80e-02 ± 4.00e-03	3.80e-02 ± 4.00e-03	6.20e-02 ± 7.63e-02	**8.80e-02** ± **2.32e-02**	7.60e-02 ± 2.42e-02
200	4.10e-02 ± 8.00e-03	3.30e-02 ± 4.00e-03	6.00e-02 ± 6.98e-02	**1.05e-01** ± **7.07e-03**	8.40e-02 ± 2.01e-02
300	4.80e-02 ± 6.18e-03	3.40e-02 ± 1.33e-03	6.40e-02 ± 5.14e-02	**1.06e-01** ± **1.32e-02**	8.47e-02 ± 1.48e-02
400	5.85e-02 ± 3.39e-03	3.50e-02 ± 2.24e-03	6.55e-02 ± 4.31e-02	**1.01e-01** ± **9.82e-03**	8.50e-02 ± 1.47e-02
500	6.16e-02 ± 5.57e-03	4.24e-02 ± 1.96e-03	7.12e-02 ± 3.94e-02	**1.02e-01** ± **9.91e-03**	8.68e-02 ± 9.68e-03
1000	7.90e-02 ± 4.34e-03	5.44e-02 ± 2.06e-03	7.06e-02 ± 2.80e-02	**9.62e-02** ± **7.83e-03**	8.54e-02 ± 4.27e-03
2000	**1.03e-01** ± **2.18e-03**	8.00e-02 ± 2.57e-03	6.78e-02 ± 2.81e-02	9.52e-02 ± 3.67e-03	8.45e-02 ± 3.73e-03

Table S37: Mean Top k edges Precision (± SD) for global GRNs against mESC_chipunion_KDUnion_intersect

Top K edges	ctOTVelo	OTVelo	SINCERITIES	GRNBoost2	scMTNI
100	**2.40e-01** ± **6.32e-03**	6.80e-02 ± 4.00e-03	8.40e-02 ± 6.53e-02	1.76e-01 ± 2.58e-02	1.46e-01 ± 3.01e-02
200	**2.35e-01** ± **4.47e-03**	1.06e-01 ± 2.00e-03	7.50e-02 ± 4.39e-02	1.48e-01 ± 1.57e-02	1.31e-01 ± 2.65e-02
300	**2.28e-01** ± **3.40e-03**	1.25e-01 ± 5.42e-03	6.00e-02 ± 3.87e-02	1.29e-01 ± 1.27e-02	1.21e-01 ± 2.20e-02
400	**2.18e-01** ± **6.40e-03**	1.27e-01 ± 4.85e-03	5.80e-02 ± 2.59e-02	1.19e-01 ± 1.01e-02	1.20e-01 ± 2.29e-02
500	**2.17e-01** ± **9.43e-03**	1.29e-01 ± 2.40e-03	5.40e-02 ± 1.80e-02	1.19e-01 ± 1.01e-02	1.20e-01 ± 2.29e-02
1000	**2.20e-01** ± **2.93e-03**	1.64e-01 ± 4.26e-03	5.04e-02 ± 9.02e-03	1.19e-01 ± 1.01e-02	1.20e-01 ± 2.29e-02
2000	**1.86e-01** ± **1.75e-03**	1.59e-01 ± 3.06e-03	5.25e-02 ± 8.81e-03	1.19e-01 ± 1.01e-02	1.20e-01 ± 2.29e-02

Table S38: Mean Top k edges Recall (± SD) for global GRNs against mESC_chipunion

Top K edges	ctOTVelo	OTVelo	SINCERITIES	GRNBoost2	scMTNI
100	**1.66e-03** ± **2.66e-05**	6.63e-04 ± 4.07e-05	6.41e-04 ± 4.14e-04	9.67e-04 ± 1.11e-04	4.54e-04 ± 8.67e-05
200	**2.54e-03** ± **2.17e-05**	1.18e-03 ± 2.17e-05	1.17e-03 ± 8.74e-04	1.65e-03 ± 1.77e-04	8.32e-04 ± 7.58e-05
300	**3.35e-03** ± **7.37e-05**	1.65e-03 ± 8.83e-05	1.96e-03 ± 1.33e-03	2.42e-03 ± 2.37e-04	1.28e-03 ± 1.65e-04
400	**4.28e-03** ± **8.69e-05**	2.66e-03 ± 9.72e-05	2.77e-03 ± 1.75e-03	3.19e-03 ± 2.80e-04	1.64e-03 ± 2.55e-04
500	**5.30e-03** ± **1.48e-04**	3.31e-03 ± 5.95e-05	3.61e-03 ± 2.18e-03	3.89e-03 ± 5.08e-04	2.02e-03 ± 2.28e-04
1000	**1.10e-02** ± **1.26e-04**	7.88e-03 ± 1.88e-04	7.51e-03 ± 4.05e-03	7.70e-03 ± 4.59e-04	3.76e-03 ± 1.68e-04
2000	**2.14e-02** ± **1.56e-04**	1.72e-02 ± 4.10e-04	1.53e-02 ± 6.09e-03	1.29e-02 ± 5.06e-04	7.18e-03 ± 2.06e-04

bioRxiv preprint doi: https://doi.org/10.64898/2026.03.11.711174; this version posted March 14, 2026. The copyright holder for this preprint (which was not certified by peer review) is the author/funder, who has granted bioRxiv a license to display the preprint in perpetuity. It is made available under aCC-BY 4.0 International license.

Table S39: Mean Top k edges Recall (± SD) for global GRNs against mESC_KDUnion

Top K edges	ctOTVelo	OTVelo	SINCERITIES	GRNBoost2	scMTNI
100	1.23e-04 ± 8.50e-06	8.08e-05 ± 8.50e-06	1.32e-04 ± 1.62e-04	**1.87e-04 ± 4.92e-05**	1.01e-04 ± 3.23e-05
200	1.74e-04 ± 3.40e-05	1.40e-04 ± 1.70e-05	2.55e-04 ± 2.97e-04	**4.46e-04 ± 3.01e-05**	2.24e-04 ± 5.36e-05
300	3.06e-04 ± 3.94e-05	2.17e-04 ± 8.50e-06	4.08e-04 ± 3.28e-04	**6.76e-04 ± 8.44e-05**	3.39e-04 ± 5.92e-05
400	4.97e-04 ± 2.88e-05	2.98e-04 ± 1.90e-05	5.57e-04 ± 3.66e-04	**8.59e-04 ± 8.35e-05**	4.54e-04 ± 7.79e-05
500	6.55e-04 ± 5.92e-05	4.51e-04 ± 2.08e-05	7.57e-04 ± 4.19e-04	**1.08e-03 ± 1.05e-04**	5.79e-04 ± 6.30e-05
1000	1.68e-03 ± 9.22e-05	1.16e-03 ± 4.38e-05	1.50e-03 ± 5.95e-04	**2.05e-03 ± 1.67e-04**	1.14e-03 ± 5.57e-05
2000	**4.39e-03 ± 9.28e-05**	3.40e-03 ± 1.09e-04	2.88e-03 ± 1.19e-03	4.05e-03 ± 1.56e-04	2.26e-03 ± 9.30e-05

Table S40: Mean Top k edges Recall (± SD) for global GRNs against mESC_chipunion_KDUnion_intersect

Top K edges	ctOTVelo	OTVelo	SINCERITIES	GRNBoost2	scMTNI
100	**1.01e-02 ± 2.66e-04**	2.86e-03 ± 1.68e-04	3.53e-03 ± 2.75e-03	7.40e-03 ± 1.08e-03	3.69e-03 ± 7.60e-04
200	**1.98e-02 ± 3.76e-04**	8.92e-03 ± 1.68e-04	6.31e-03 ± 3.70e-03	1.25e-02 ± 1.32e-03	6.62e-03 ± 1.34e-03
300	**2.88e-02 ± 4.29e-04**	1.58e-02 ± 6.84e-04	7.57e-03 ± 4.88e-03	1.61e-02 ± 1.79e-03	8.04e-03 ± 1.05e-03
400	**3.67e-02 ± 1.08e-03**	2.14e-02 ± 8.16e-04	9.76e-03 ± 4.35e-03	1.64e-02 ± 2.01e-03	8.04e-03 ± 1.05e-03
500	**4.57e-02 ± 1.98e-03**	2.71e-02 ± 5.05e-04	1.14e-02 ± 3.78e-03	1.64e-02 ± 2.01e-03	8.04e-03 ± 1.05e-03
1000	**9.26e-02 ± 1.23e-03**	6.89e-02 ± 1.79e-03	2.12e-02 ± 3.80e-03	1.64e-02 ± 2.01e-03	8.04e-03 ± 1.05e-03
2000	**1.56e-01 ± 1.47e-03**	1.34e-01 ± 2.57e-03	4.42e-02 ± 7.42e-03	1.64e-02 ± 2.01e-03	8.04e-03 ± 1.05e-03

Table S41: Mean Top k edges Number of predictable TFs (± SD) for global GRNs against mESC_chipunion

Top K edges	ctOTVelo	OTVelo	SINCERITIES	GRNBoost2	scMTNI
100	**1.00e+00 ± 0.00e+00**	**1.00e+00 ± 1.73e+00**	2.00e-01 ± 4.00e-01	0.00e+00 ± 0.00e+00	2.00e-01 ± 4.00e-01
200	**1.00e+00 ± 0.00e+00**	4.00e-01 ± 7.35e-01	6.00e-01 ± 8.00e-01	0.00e+00 ± 0.00e+00	2.00e-01 ± 4.00e-01
300	**1.00e+00 ± 0.00e+00**	6.50e-01 ± 1.15e+00	6.00e-01 ± 8.00e-01	0.00e+00 ± 0.00e+00	2.00e-01 ± 4.00e-01
400	8.00e-01 ± 4.00e-01	7.50e-01 ± 1.30e+00	**1.20e+00 ± 7.48e-01**	0.00e+00 ± 0.00e+00	2.00e-01 ± 4.00e-01
500	2.00e-01 ± 4.00e-01	8.00e-01 ± 1.40e+00	**1.20e+00 ± 7.48e-01**	0.00e+00 ± 0.00e+00	2.00e-01 ± 4.00e-01
1000	1.00e+00 ± 0.00e+00	1.05e+00 ± 1.83e+00	**1.20e+00 ± 9.80e-01**	0.00e+00 ± 0.00e+00	0.00e+00 ± 0.00e+00
2000	1.00e+00 ± 0.00e+00	1.40e+00 ± 2.01e+00	**2.20e+00 ± 1.17e+00**	2.00e-01 ± 4.00e-01	0.00e+00 ± 0.00e+00

Table S42: Mean Top k edges Number of predictable TFs (± SD) for global GRNs against mESC_KDUnion

Top K edges	ctOTVelo	OTVelo	SINCERITIES	GRNBoost2	scMTNI
100	0.00e+00 ± 0.00e+00	**1.00e+00 ± 1.73e+00**	0.00e+00 ± 0.00e+00	0.00e+00 ± 0.00e+00	0.00e+00 ± 0.00e+00
200	0.00e+00 ± 0.00e+00	**4.00e-01 ± 7.35e-01**	**4.00e-01 ± 4.90e-01**	0.00e+00 ± 0.00e+00	0.00e+00 ± 0.00e+00
300	0.00e+00 ± 0.00e+00	**6.50e-01 ± 1.15e+00**	2.00e-01 ± 4.00e-01	0.00e+00 ± 0.00e+00	0.00e+00 ± 0.00e+00
400	0.00e+00 ± 0.00e+00	**7.50e-01 ± 1.30e+00**	2.00e-01 ± 4.00e-01	2.00e-01 ± 4.00e-01	0.00e+00 ± 0.00e+00
500	0.00e+00 ± 0.00e+00	**8.00e-01 ± 1.40e+00**	4.00e-01 ± 4.90e-01	2.00e-01 ± 4.00e-01	0.00e+00 ± 0.00e+00
1000	0.00e+00 ± 0.00e+00	**1.05e+00 ± 1.83e+00**	4.00e-01 ± 4.90e-01	2.00e-01 ± 4.00e-01	0.00e+00 ± 0.00e+00
2000	8.00e-01 ± 4.00e-01	**1.40e+00 ± 2.01e+00**	4.00e-01 ± 4.90e-01	2.00e-01 ± 4.00e-01	0.00e+00 ± 0.00e+00

Table S43: Mean Top k edges Number of predictable TFs (± SD) for global GRNs against mESC_chipunion_KDUnion_intersect

Top K edges	ctOTVelo	OTVelo	SINCERITIES	GRNBoost2	scMTNI
100	0.00e+00 ± 0.00e+00	**1.00e+00 ± 1.73e+00**	0.00e+00 ± 0.00e+00	0.00e+00 ± 0.00e+00	0.00e+00 ± 0.00e+00
200	0.00e+00 ± 0.00e+00	**4.00e-01 ± 7.35e-01**	0.00e+00 ± 0.00e+00	0.00e+00 ± 0.00e+00	0.00e+00 ± 0.00e+00
300	0.00e+00 ± 0.00e+00	**6.50e-01 ± 1.15e+00**	0.00e+00 ± 0.00e+00	0.00e+00 ± 0.00e+00	0.00e+00 ± 0.00e+00
400	0.00e+00 ± 0.00e+00	**7.50e-01 ± 1.30e+00**	0.00e+00 ± 0.00e+00	0.00e+00 ± 0.00e+00	0.00e+00 ± 0.00e+00
500	0.00e+00 ± 0.00e+00	**8.00e-01 ± 1.40e+00**	0.00e+00 ± 0.00e+00	0.00e+00 ± 0.00e+00	0.00e+00 ± 0.00e+00
1000	0.00e+00 ± 0.00e+00	**1.05e+00 ± 1.83e+00**	0.00e+00 ± 0.00e+00	0.00e+00 ± 0.00e+00	0.00e+00 ± 0.00e+00
2000	0.00e+00 ± 0.00e+00	**1.40e+00 ± 2.01e+00**	2.00e-01 ± 4.00e-01	0.00e+00 ± 0.00e+00	0.00e+00 ± 0.00e+00
